# Optical Monitoring of Microplastics Filtrated from Wastewater Sludge and Suspended in Ethanol

**DOI:** 10.3390/polym13060871

**Published:** 2021-03-11

**Authors:** Benjamin O. Asamoah, Pauliina Salmi, Jukka Räty, Kalle Ryymin, Julia Talvitie, Anna K. Karjalainen, Jussi V. K. Kukkonen, Matthieu Roussey, Kai-Erik Peiponen

**Affiliations:** 1 Department of Physics and Mathematics, University of Eastern Finland, P.O. Box 111, FI-80101 Joensuu, Finland; matthieu.roussey@uef.fi; 2 Faculty of Information Technology, University of Jyväskylä, Mattilanniemi 2 (Agora building), P.O. Box 35, FI-40014 Jyväskylä, Finland; pauliina.u.m.salmi@jyu.fi; 3 Unit of Measurement Technology, MITY, University of Oulu, Technology Park, P.O.BOX 127, FI-87400 Kajaani, Finland; jukka.raty@oulu.fi; 4 Department of Biological and Environmental Science, University of Jyväskylä, Survontie 9C (YAC Building), P.O. Box 35, FI-40014 Jyväskylä, Finland; kalle.ryymin@gmail.com (K.R.); anakakarjalainen@gmail.com (A.K.K.); 5 Marine Management, Finnish Environment Institute (SYKE), Latokartanonkaari 11, FI-00790 Helsinki, Finland; julia.talvitie@syke.fi; 6 Department of Environmental and Biological Science, University of Eastern Finland, Kuopio Campus, P.O. Box 1627, FI-79211 Kuopio, Finland; jussi.kukkonen@uef.fi

**Keywords:** microplastics, sludge, wastewater, Raman spectroscopy, laser speckle pattern, transmittance, sedimentation

## Abstract

The abundance of microplastics (MPs) in the atmosphere, on land, and especially in water bodies is well acknowledged. In this study, we establish an optical method based on three different techniques, namely, specular reflection to probe the medium, transmission spectroscopy measurements for the detection and identification, and a speckle pattern for monitoring the sedimentation of MPs filtrated from wastewater sludge and suspended in ethanol. We used first Raman measurements to estimate the presence and types of different MPs in wastewater sludge samples. We also used microscopy to identify the shapes of the main MPs. This allowed us to create a teaching set of samples to be characterized with our optical method. With the developed method, we clearly show that MPs from common plastics, such as polypropylene (PP), polyethylene terephthalate (PET), polystyrene (PS), and polyethylene (PE), are present in wastewater sludge and can be identified. Additionally, the results also indicate that the density of the plastics, which influences the sedimentation, is an essential parameter to consider in optical detection of microplastics in complex natural environments. All of the methods are in good agreement, thus validating the optics-based solution.

## 1. Introduction

The recent increase in publications shows that research on microplastics (MPs) is a topic of utmost importance for the environment and health despite the recent emergence and recognition of the problem by the scientific community [1,2,3,4,5,6,7]. The multi-disciplinary character of the topic makes the homogenization of the research complex. Although there are still some inconsistencies in the sampling methods [8] and sample processing of the microplastics, as well as in the analysis methods, a trend can be seen in the uniformity of the techniques. Another study emphasized the issue of discrepancies between plastic particle types, size ranges, and concentrations in tests that were carried out either in a laboratory or in real aquatic environments [1]. According to Alexy et al. [1], there is a need to harmonize the methods for better comparability of the results as well as a requirement to have automated sensors for real-time and/or continuous measurements and data processing.

Many analysis methods and techniques have been developed for the identification of MPs [9,10]. Through Fourier-transform-infrared (FTIR) [11] and Raman [12] spectroscopies, optics are widely used, although these two techniques still require sample harvesting and preparation prior to measurements. Regarding the automated and real-time detection of microplastics, optical methods are among the most powerful alternatives and offer many different opportunities based on well-established concepts. However, there is often the remaining problem of sampling microplastics, which are usually required in the form of dry samples for measurements [13].

Plastics can be identified using the spectral fingerprints of synthetic hydrocarbons in the near-infrared (NIR) spectral range. Applying spectral techniques in this wavelength range in the detection of plastics in an aquatic environment is rather complicated due to the strong NIR absorption by water, which interferes with the method of plastic detection and identification. To navigate this problem, dry sample measurements are, therefore, preferred. However, in the vicinity of λ = 1200 nm, water absorption is weaker, and methods such as airborne detection of floating microplastics have been suggested, but for harvested samples only [14]. An important step forward for understanding the behavior of microplastics in aquatic environments using optical sensing is to realize in situ and real-time measurement methods that require no harvesting or sample treatment of any kind. This is a challenging task, however, due to the relatively low concentration of MPs—with partial or full transparency in some cases—as well as the complex and changing environments. Moreover, most of the current optical techniques for MPs are powerful when used separately. However, for an accurate identification, quantification, and classification of MPs, the development of a multifunctional sensor based on the clever integration of the individual optical methods is necessary for in situ monitoring. Therefore, simulations and tests on different types of optical phenomena in controlled laboratory conditions are needed to progress in the design and development of such in situ optical detectors.

In this paper, we combine spectroscopic methods based on transmittance and laser-based measurements to identify and validate MP detection in a complex matrix. The method is verified with Raman measurements. We demonstrate that this approach is a promising tool in the detection of low concentrations of different MPs in complex environments and for implementation in real-time measurements. Moreover, the study validates the technique, which could serve as a qualitative control in wastewater treatment processes in order to monitor the MPs after treatment [13,15]. The microplastics were obtained from wastewater sludge samples and suspended in ethanol (filtrated and suspended in ethanol MPs: FEMPs). Ethanol, like water, has a spectral fingerprint in the NIR region and, therefore, presents similar challenges for in situ optical detection of MPs in aquatic environment. In addition, ethanol improves the measurement safety, as it kills harmful bacteria and viruses [16], which can exist in wastewaters.

Detecting particles in wastewater sludge is easy; detecting one specific type of particle is the key challenge of current research in this field. To perform such an analysis in the field, we have established a set of samples to be used to learn the behavior of the MP particles coming from such a harsh environment. The outcomes of the investigations on this teaching sample set with our newly developed optical method(s) can then be used in real conditions, i.e., outside the laboratory, and can serve as a basis for the determination of a new detection methodology. The goal is, therefore, to first determine the presence of MPs in wastewater samples through Raman spectroscopy, as well as their shape through microscopy, in order to establish our teaching test set. Further, we used this teaching set to determine whether our optical methods are suitable for detecting the same MPs in the same samples.

## 2. Materials and Methods

In this section, we give a detailed description of the sample preparation and optical detection schemes that could be an alternative to the time-consuming Raman spectroscopy detection method for MPs. However, as a reference for a comparative study, we use data from a Raman spectrometer that was exploited in the identification of MPs of different plastic types that originated from a municipal wastewater plant. The advantage of the different proposed optical method(s) is the relatively quick measurement time and the ease of combining them in a multi-functional optical device to be used for quality inspections in wastewater treatment.

### 2.1. Sample Preparation

To obtain the microplastics (20–5000 µm), cake samples of anaerobically digested and dried wastewater sludge from the Nenäinniemi wastewater treatment plant (Jyväskylä, Finland) were collected in aluminum foil and stored at +4 °C in 1 L plastic boxes. Subsamples of 40–42 g wet weight (11–14 mg dry weight) were purified with hydrogen peroxide (30%, WVR International) and enzymes (ASA Special enzyme GmbH, Wolfenbüttel, Germany) in 250 mL glass bottles according to the universal purification protocol developed and validated in [17]. The final step in the protocol was the density separation of microplastics with ZnCl_2_ solution (ρ = 1.6–1.8 g cm^−3^). Before the purification protocol, hydrogen peroxide and the enzymes were pre-filtered through GF/A filters (Whatmann, Maidstone, UK) to remove possible microplastic contaminants. The ZnCl_2_ solution was filtered through a 20 µm stainless-steel filter because it easily blocked the GF/A filters.

The high dry matter content of our samples prevented the direct application of the vacuum filtration described by Löder et al. (2017) [17]. Instead, we washed the samples by centrifugation between each step as follows: Samples were poured in 50 mL centrifuge tubes, covered with foil, and centrifuged at 3000 rcf for 2 min. The supernatant was then filtered using 20 µm mesh-sized stainless-steel filters. The remaining pellet was again centrifuged using ultrapure water to clean the sample, and the supernatant was filtered. The filter and the pellet were transferred to the 250 mL glass bottle and submerged with the reagent of the next step. Another modification introduced to the method of Löder et al. (2017) was the replacement of sodium dodecyl sulfate (SDS) in step 1 with 30% hydrogen peroxide due to the extensive foaming of SDS. Hydrogen peroxide incubations were done at 60 °C.

**Figure 1:**
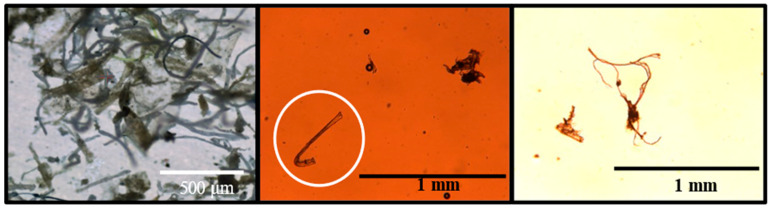
Micrograph of microplastics (MPs) during preparation (left) with different shapes and sizes and those identified in the measured sample (middle). In the white circle, a hook-shaped MP is highlighted, and the other black spots are air bubbles and unidentified organic materials (also in the right image).

Finally (step 8, Table 1), the microplastics were rinsed with ZnCl_2_ (ρ = 1.6–1.8 g.cm^−3^) from the steel filter into a glass separation funnel. Sand and other relatively heavy materials were separated by incubating for 1–3 days. Microplastics remaining in the ZnCl_2_ were collected on the steel filter and eluted with ethanol (70% in water) in glass bottles for storage. The resulting mixture was a diluted concentration of different MPs filtrated and preserved in ethanol (FEMPs). Figure 1 shows images of the MPs during the preparation process (left) and contained in the measured samples (in the white circle, middle). One can appreciate their various sizes and irregular shapes, e.g., “hook-like”, fibers, fragments, and aggregates. Despite the filtering process, other unidentified organic materials than MPs were clearly present (right) after preparation. Such particles are typical of wastewater and natural environments, and they add to the complexities of the matrix. More information and details are given in the dataset published by P. Salmi et al. [18].

### 2.2. Counting and Characterization of Microplastics with Raman Microscopy

Subsamples of 5 mL of the FEMP samples were filtered on aluminum oxide filters (Anodisc, Whatmann, Maidstone, UK). Microplastics were manually counted on at least 15% of the area of the filter, and the plastic type was analyzed from the field of view of a Raman microscope (Thermo Fischer Raman DXR 2xi, Waltham, MA, USA). Table 2 gives the numbers of particles and subsampling ratios. The Raman spectra of microplastics were obtained with 758 nm laser excitation (20 mW power), an objective with ×10 magnification, a 50 µm slit aperture, and an exposure time of 0.1 s for 20 scans. The acquired spectra were compared to commercial material libraries using the OMNIC xi software (Thermo Fischer Scientific, Waltham, MA, USA). For pristine samples, the match to library spectra was expected to be higher than 70%; however, due to matrix interference, a 50–70% match was obtained based on the Raman peak positions. The particles characterized as plastic were categorized as fragments or fibers based on the deviation from the sting-shaped form of the fibers. Counts were converted to microplastics per gram of dry weight (MP g^−1^ DW) according to Equation (1).

*MP g*^−1^*DW* = [(*N* × *k*) / *m*] × *df*, (1)

where *N* is the number of particles counted, *k* is the ratio of the filtration area to the area where the particles were counted from, *m* is the dry mass of the processed sample, and *df* is the dilution factor because the whole processed sample was not filtered with the aluminum oxide filter.

### 2.3. Three Optics-Based Alternatives for a New Detection Method

The same samples were also characterized—benchmarking with ethanol—with three other devices to compare with the results of Raman spectroscopy and to determine the dynamic light scattering of the identified MPs. The devices were (1) a spectrophotometer (UV 3600, Shimadzu, Kyoto, Japan) for obtaining the spectral fingerprints, (2) a handheld diffractive optical element (DOE, MGM Devices, Finland)-based optical sensor [19], and, finally, (3) a “basic” optical setup consisting of a Helium–Neon (HeNe) laser light source (5 mW, λ = 632.8 nm) and a charge-coupled device (CCD, Thorlabs, Newton, NJ, USA) camera for probing the samples. Both the basic setup and DOE-based sensors were used for the determination of the dynamic light scattering properties of the FEMP samples.

**Figure 2:**
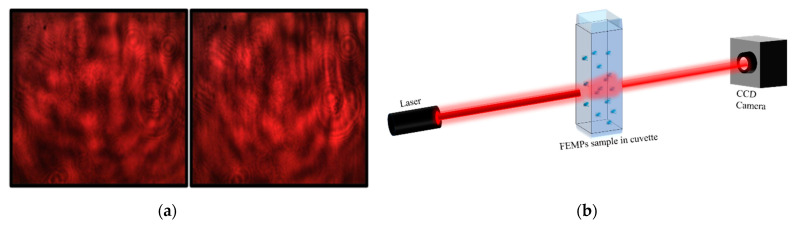
(**a**) Speckle pattern of samples on a rough glass surface for samples of ethanol (left) and MPs filtrated and preserved in ethanol (FEMPs) (right). The recorded static images (patterns) are very similar to the naked eye for both samples. (**b**) Basic optical experimental setup for recording the beam profile after propagation through the FEMP sample.

One of our novel methods is based on the modification of an original handheld glossmeter to create a DOE-based optical sensor. A detailed description of the device is given in [19]. It consists of a handheld glossmeter with a single-cell photodiode for recording the specular reflection and an attached CCD camera—without an objective lens—for recording the forward-scattered light. Moreover, for liquid samples, there is a compartment designed with an 800 μL inner volume to contain the liquid for measurement. The liquid compartment consists of a glass disk base (with either both sides smooth or one side rough) and a black surrounding cup. The use of the rough glass disk enables the generation of a speckle pattern—see Figure 2a—with a coherent laser light source for probing liquid-based samples. The laser speckle pattern is formed due to coherent laser light scattering from microscopic objects, such as MPs in water or in other host liquids. This pattern is sensitive to the local displacement of particles, i.e., sinking or moving in the direction of the beam. The presence of MPs is, therefore, expected to modify this original speckle pattern, leading to the detection of the presence of the MPs. The extent of the speckle pattern modification by the MPs depends on the properties of the MPs, such as the size, volume properties, and surface texture [20,21], as well as the refractive index (RI) mismatch between the present material and the ambient medium. For very small MP sizes, as considered in this study, few modifications to the speckle and interference patterns are expected. Replacing the rough disk with smooth glass leads to the transmission of an interference pattern; see [19]. Connecting the DOE-based sensor, which is equipped with a semiconductor laser light source (0.8 mW, λ = 635 nm), to a computer enables time-dependent measurements [22], which may be essential for studies in the field.

The speckle contrast [23], *C = [< I^2^ > − < I >]^1/2^ / < I >*, was calculated to obtain information on the dynamics of the samples as a result of the modification to the initially generated speckle pattern with time. *I* and *< ^…^>.* represent the intensity of the speckle pattern and the ensemble average, respectively. The speckle contrast was calculated using the intensity of the red channel from the red–green–blue (RGB) pattern captured on the CCD camera after correcting with the measured stray light. *C* has values ranging from 0 to 1 for undeveloped and fully developed speckles, respectively. It has a long history of applications ranging from surface roughness characterization [21,24] to flow rate determination [25]. In this study, we employed it in the qualitative identification of the presence of microplastics. The data obtained with these optical methods, which are presented in Figure 2, will be treated in the last part of the manuscript while describing the detection of the sedimentation of MPs.

### 2.4. Measurement Procedure with the Spectrophotometer and the New Optical Devices

First, the transmittance of the FEMPs and the reference ethanol samples was measured with the spectrophotometer in the visible-near-infrared (Vis-NIR) spectral range of 500–2500 nm, where most plastics have spectral fingerprints [26,27,28]. Additionally, a standard spectroscopic cuvette with inner dimensions of 10 mm × 5 mm × 35 mm and an inner volume of ca. 1750 µL was filled with the FEMP sample. The cuvette was set so that light propagated along the 5 mm direction in the sample. The transmittance as a function of time at a fixed wavelength *λ* = 800 nm was then measured after shaking the sample in the cuvette. The settling of the MPs after shaking was to simulate the sedimentation process of the different MPs due to variation in densities. In both measurements, an empty cuvette was used for the baseline measurements.

Next, the “basic” laser-based optical setup was used to measure the pure ethanol and the FEMP samples in another standard spectroscopic quartz cuvette with dimensions of 10 mm × 10 mm × 35 mm (ca. 3500 µL). The covered cuvette was shaken and illuminated with a Helium–Neon (HeNe) laser light source with a 0.81 mm beam diameter. The corresponding transmitted beam was also recorded with time on a CCD camera—also without an objective lens—placed 60 cm from the cuvette to determine the dynamic laser-light-scattering properties of the particles in the FEMP sample.

Lastly, using the smooth glass disk base in the modified portable handheld device, we introduced a 15 μL drop of the filtrated sample into the volume compartment of the DOE-based sensor with a glass pipette to measure the specular reflection of the sample with time. Since there was very little probability of having any MPs in such a small volume, we were only able to obtain information on the effective optical medium of the FEMP sample after the preparation process based on the specular reflection. To further probe the dynamic light-scattering properties of the samples in a relatively larger volume, using the same device but with a rough glass disk base, the 800 μL (for an approximate height of 2.8 mm and glass disk diameter of 20 mm) volume compartment was filled with 700 μL of the pure ethanol and the FEMP sample, and the transmitted pattern was again measured with time on the CCD camera. Static images of the recorded speckle patterns are shown in Figure 2a. It is worth mentioning that the images are very similar for both the ethanol and FEMP samples. This optical method simultaneously detected the presence of various types and sizes of mostly irregularly shaped MPs in the liquid matrix.

For comparison, we also performed similar measurements for pure ethanol (96%), in which the FEMPs were preserved. We noted that the speckle pattern measurements with the DOE-based sensor and the HeNe laser setup differed. These gave different transmittance schemes and possibly different numbers of particles in the considered sample volumes. However, both methods showed similar dynamic activity of the FEMP samples. The probing laser light beam was limited by an aperture of 2 mm at the bottom of the glass disk in the DOE-based sensor. In the same device, the FEMP samples were introduced directly on the light beam’s path. Hence, the motion of the FEMPs was preferentially falling parallel to the direction of the beam, whereas in the other schemes, the FEMPs were falling orthogonal to it. Therefore, in the case of the recorded speckle pattern, the DOE-based sensor monitored variations in the speckle pattern with time because of local variations in the surface roughness of the glass disk base.

It is to be noted that the use of the spectrophotometer (detection of the transmission spectrum) prevents its use for several minutes or seconds for other purposes, such as monitoring the sedimentation. For this reason, we needed a laser-based sensor that, thanks to coherent radiation, was very sensitive in order to detect any small changes along the path of the laser beam, e.g., an MP passing in the beam.

## 3. Results and Discussion

### 3.1. Raman Microscopy

The analysis of the Raman microscopy spectra led to the identification of the following MPs in the FEMP sample: polypropylene (PP), polyethylene (PE), polyethylene terephthalate (PET), polystyrene (PS), and polyoxymethylene (POM). The numbers of MPs counted for each shape of the identified plastic types are presented in Table 2 for the three sets of FEMP samples that were measured (Samples A, B, and C). The corresponding estimated concentrations of the MPs (mean = 17 MPs/mL, SD = 2) from the Raman measurements are indicated for the samples. The average densities of the identified MPs are also presented in the same table to aid in a later discussion of the other results. By comparing the measured Raman spectra to the commercial material library, MPs of five (5) different plastic types could be identified in the FEMP samples, which is in accordance with the most abundant MPs in wastewater [29].

Raman spectroscopy provided a clear picture of the nature and types of MPs in the samples to be investigated with our novel optical method. First, one can remark that PET fibers are predominant. Second, the number of fibers is negligible for PP, PS, and POM. No particles, but only fibers and in a very small amount, were detected for PE.

These results, which were obtained with a recognized method for MP identification, allowed us first to determine the presence and types of MPs in the prepared samples, second, to establish a set of known samples obtained from a wastewater treatment plant, and, third, to further validate our simpler optical methods.

### 3.2. Specular Reflection Signal

**Figure 3:**
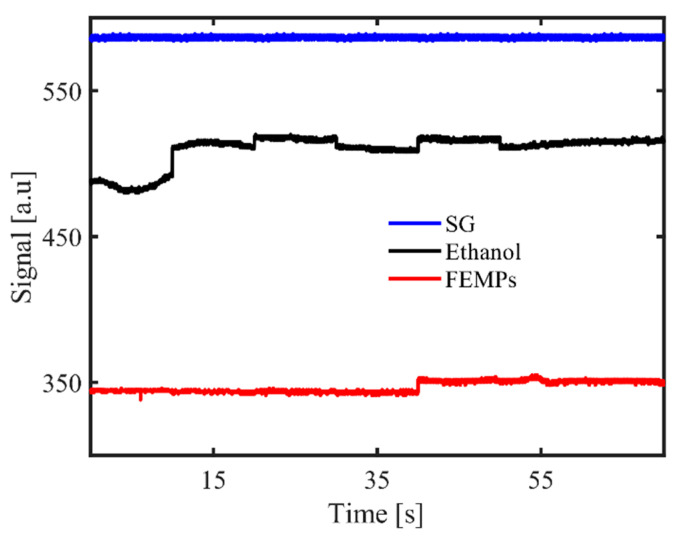
Specular reflection recorded with the diffractive optical element (DOE)-based sensor for the smooth glass (SG) disk base only for the pure ethanol (Ethanol) and FEMP (MPs preserved in ethanol) samples.

Figure 3 shows the reflection signal detected with the DOE-based sensor after introducing 15 μL of the FEMP sample on the smooth glass disk base. The recorded specular reflection signal had an accuracy of ca. 0.10%. We observe that both the pure ethanol and FEMP samples showed lower reflection signals than with the smooth glass only, with that of pure ethanol being higher than that of the FEMP sample. The clear difference in the magnitudes of the reflection signals between the pure ethanol and FEMP samples was more likely due to the difference in the refractive indexes of the two samples. Pure ethanol has a refractive index (RI) of n = 1.36; by preserving the filtrated sample in ethanol, we changed the effective refractive index of the resultant mixture, which yielded a change in the reflection signal. Obtaining a lower reflection signal than that of pure ethanol indicates that the FEMP sample had a higher effective RI than the pure ethanol. However, we note that, by measuring the specular reflection of such a small volume on the smooth glass, we obtained information on the ambient medium of the FEMP sample rather than on the MPs, the reason being that such a small volume of the FEMP sample has very little probability of containing an MP, although it may contain other particles (see Figure 1) that also affect the effective RI. Additionally, considering the fairly constant nature of the reflection signal with time, one can conclude that both liquids rarely spread over the smooth glass [22,30]. This is especially true for the FEMP sample due to its slightly more viscous nature than that of the pure ethanol.

### 3.3. Transmission Spectra

**Figure 4:**
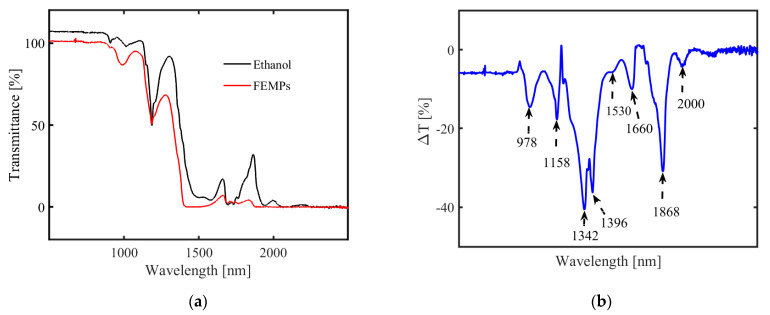
Transmission spectra of (**a**) pure ethanol (black curve) and the FEMP (MPs preserved in ethanol, red curve) samples and (**b**) differences between the two spectra (ΔT).

The transmission spectra of pure ethanol and the FEMPs are shown in Figure 4a. From the Raman measurements, we know that FEMP samples A, B, and C contained five different plastic types. The measured transmittance is, therefore, a combination of their weighted spectral features. For λ < 900 nm, both samples showed T > 100%, which is due to the use of air for the baseline correction. Pure ethanol showed a higher transmittance than the FEMP samples across almost all wavelengths, with dominant features for λ > 1100 nm. The magnitude of MP transmittance in a liquid medium is highly dependent on the concentration [26]. To identify and classify the different MPs in the FEMP samples based on the spectral features, we subtracted the transmittance of pure ethanol from that of the FEMP samples. Figure 4b shows the resulting difference in transmittance, ΔT, where the spectral features, seen as dips, consisting of first-third overtones of C–H vibrations [31], are more visible. In comparison to earlier studies [26,27,28], these observed spectral features can be attributed to MPs from known plastic types. In the study by Peiponen et al. (2019) [26], where the spectral features of plastics were studied in both air and water, it was observed that the maximum deviation of an identified absorption peak is about 4 nm, despite the relatively large difference in RI mismatch. In some cases, no deviations were observed for some peaks of the same plastic type.

Imposing similar constraints and considering the small difference in the dispersion of ethanol and water, we can therefore attribute some of the identified spectral dips as follows: 1158 (PS), 1396 (PE), and 1660 nm (PET). This identification and classification, such as in the case of PET, is in close agreement with the results of [28], who performed spectral measurements of plastics in air at NIR and mid-infrared. As is evident from [28], POM shows no spectral features in the Vis-NIR spectral range; hence, none of the fingerprints can be attributed to it, but due to its density, it will contribute to the sedimentation process. Based on these observations, we can classify the mixture of MPs by simply combining transmittance measurements with an a priori library of MP spectral properties, such as that presented in [28]. The creation of such a rich library of intrinsic spectral properties of plastics could also be beneficial in quantifying the concentrations of the different plastic types using chemometric tools, such as principal component analysis [31]. However, we remark that for real-time monitoring and detection of plastics in open water, the problem of suppressing the spectral fingerprints of water absorption could persist [26]. The unattributed dips at 978, 1530, and 1868 nm can be due to the water content present in the preserving ethanol (ca. 30%).

Thus far, based on the transmission and the Raman measurements, similar types of MPs could be identified in the FEMP sample—excluding the POM, which showed no spectral features in the measured range. Although the shapes of the identified MPs could not be distinguished in the transmission measurements, combining both techniques in a sensor promises a more quantitative means of identifying and validating detected MPs in natural environment with higher confidence.

### 3.4. Sedimentation

**Figure 5:**
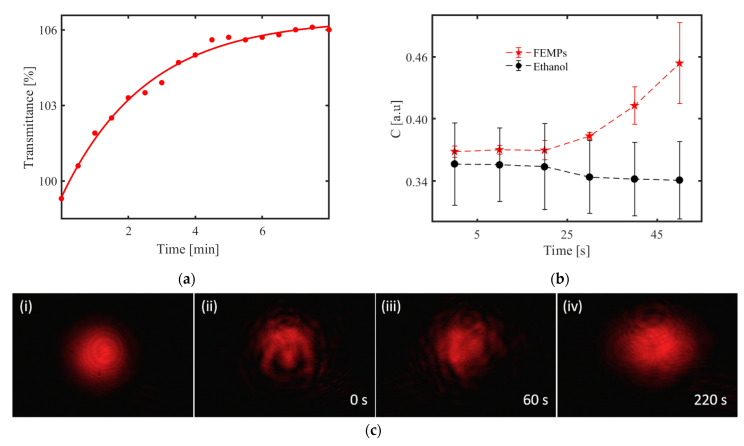
Sedimentation: (**a**) Measured transmittance (red dots) of the FEMP (MPs preserved in ethanol) samples with time at λ = 800 nm. The red curve is an exponential fit of the data. (**b**) Calculated speckle contrast from the measured speckle pattern using the diffractive optical element (DOE)-based sensor with a rough glass disk base and 700 μL volume of the samples. (**c**) Recorded patterns of the light beam transmitted through (i) pure ethanol and FEMPs after shaking at (ii) 0, (iii) 60, and (iv) 220 s with the charge-coupled device (CCD) camera.

We consider the case of MP sedimentation from the three measurement schemes, where the densities of the MPs play a major role. Figure 5a illustrates the evolution of the transmittance with the time measured with the spectrophotometer when the light beam passed through the shaken liquid. Figure 4a shows that the probing wavelength selected for the sedimentation investigations, *λ* = 800 nm, ensures that the measured transmittance of the FEMP sample is predominantly due to the scattering and diffraction (depending on the polydisperse particles) rather than absorption. The red dots are the measured transmittance with time, whereas the red curve is the exponential fit of the data points. Similarly, the observed *T* > 100%, which is due to the use of air for the baseline correction, still exists. It is obvious that the transmittance increases as a function of time. This is attributed to the sedimentation of the MPs in ethanol. Ethanol has a relatively lower density of 0.81 g cm^−3^, and the densities of the MPs in the FEMPs suggest that they will settle at different depths. However, after shaking the cuvette, the FEMPs are first in a dynamic state before starting to settle, depending on the densities. Based on the densities, most of the MPs will settle at the bottom, leaving fewer particles of PS and some available organic materials with lower density to interact with the probing light after some time has elapsed. Indeed, the curve tends to saturate after 4 min. Thus, the exponential growth of the curve is strongly influenced by the types of particles available.

Next, we consider the case of DOE-based sensor with the full volume (700 μL). The recorded speckle patterns of the pure ethanol and FEMP samples are very similar and indistinguishable to the naked eye, as seen in Figure 2a. However, due to the sensitivity of the speckle pattern to the local variation and the polydisperse particle sizes, the little variations in the pattern may be revealed in the speckle contrast, *C*.

Figure 5b shows the speckle contrast, with error bars, calculated from the recorded speckle pattern using the DOE-based sensor with a rough glass disk base for the case of 700 μL of pure ethanol and FEMP samples. We observed a fairly constant mean value of *C* with time in the case of pure ethanol, whilst a similar nonlinear relationship, as seen in Figure 5a, exists between the mean of *C* and the time for the FEMP sample. The larger variation in *C* for the pure ethanol could be due to its volatility over longer durations, which is not the case for the FEMP slurry sample. In the absence of any particles in pure ethanol, the scattering is influenced by the RI difference between the (rough) glass and the ethanol, which does not change with time; hence, the horizontal nature of the speckle contrast is as one would expect. However, the FEMP sample is rather complicated, as the scattering, in addition to the RI of the ambient medium, is also influenced by the presence, sizes, and irregular shapes of the MPs and their refractive indices. The different RI values of the MPs cause local changes in the RI for the probing beam, thus modifying the speckle pattern at the detection plane. Further increases in the speckle contrast can also be because of the falling of heavier MPs, such as POM, PS, PET, and PE, and random movement of less dense MPs, such as PP, thus modifying the speckle pattern in similar manner to that found when increasing the surface roughness. In such a small height of 2.8 mm, the speckle contrast tends to be sensitive to such sedimentation dynamics of the particles. Considering the speckle pattern as a transmission component of the DOE-based sensor, the positions of the specular reflection signals and the speckle contrast for the ethanol and the FEMP samples are in good agreement.

Comparing the integrated transmittance in Figure 5a and the speckle contrast curves in Figure 5b, we observe that both display dynamic activity in the system, which can be attributed to the presence of the MPs in the FEMP sample. However, the integrated transmittance curve saturates at a much longer time (after 4 min), which could be due to the different shapes of the respective containers. In addition to the possible higher number of MPs with the increased sample volume, the height of the cuvette (35 mm) and the area of the sensing beam (3 × 8 mm^2^) contribute to the longer saturation time in the case of the transmittance measurements, unlike in the DOE-based sensor with a 2.8 mm height of the volume compartment. Regarding the simultaneous presence of both regularly and irregularly shaped MPs with different densities, the particles are likely to spend a longer time in the narrow cuvette, leading to a longer saturation time than that for the wider cup of the DOE-based sensor with a much shorter sedimentation height.

The images in Figure 5c were obtained using the optical setup described in Figure 2b. Figure 5c shows static images related to similar measurements, but using the HeNe laser beam and a 10 mm cuvette. In Figure 5c (i), the pattern recorded for pure ethanol, which was stable for various durations, is shown. We observe regular and symmetric annular interference fringes, which could result from the thermo-optic properties of ethanol and the Gaussian intensity profile of the HeNe laser [32,33,34]. The presence of the annular rings serves as a measure of the extent of distortion of the incident beam by the FEMP sample. Figure 5c (ii) and (iii), on the other hand, show an obviously distorted beam even after 60 s of shaking the cuvette. The distortion of the beam evolves with time. The pattern, however, becomes steady after 220 s, as shown in Figure 5c (iv) with secondary circular rings. Although the macroscopic patterns in Figure 5c (i) and (iv) resemble each other, we assume that the haziness is due to the scattering of light from small particles—possibly micrometer-sized MPs. The patterns taken at 0 and 60 s show that the beam interacted with some particles, which we attribute to the MPs due to the low concentration of the organic matter present in the FEMP sample.

The amplitude of the distortion can be greatly influenced by the properties of the particles, such as the size, number, shape, and surface texture, interacting with the beam. For example, a relatively large particle compared to the beam diameter with some surface roughness can lead to a speckle pattern. In principle, smaller particles can also lead to edge diffraction and interference fringes for thin and long particles [35,36]. Considering the different and complex shapes and sizes of the MPs in the micrograph (Figure 1a), one would expect non-conventional light scattering, resulting in the complex combination of the above-stated optical phenomena, to be at play, with no particularly dominant phenomenon as observed in the 0 and 60 s patterns. In both cases of the transmittance (with time) and beam profile measurements, we observed the effect of the density of the MPs, as well as their relative positions during the sedimentation process with respect to the probing beam’s position. In general, the sedimentation process of MPs in a natural aquatic environment can be complex, as the density of plastics is influenced by aging [37,38] and the flow of water, which is constantly changing. It is, therefore, evident that in the design of an optical sensor for the detection of MPs in their natural environments, such as rivers and lakes, where the situation in constantly changing, the density of the particles should be taken into consideration. This can be done by combining the measurements of the intensity and size of the speckle in addition to the contrast [39]. All variations are nonlinear, but they can be detected with optical sensors through a referencing scheme. The movement as well as the nature of the plastic can thus be determined.

### 3.5. Discussion of the Methods

Raman microscopy allowed us to determine the different plastic types present in the samples with high precision. The first step in our optical method(s) is to examine the optical medium of the sample with specular reflection after the filtration and suspension in ethanol to determine whether we can detect a change in the samples due to the sample preparation. This is confirmed by the results presented in Section 3.2. The results from the transmission spectroscopy of MPs in the liquid are in good agreement with those of the Raman spectroscopy, since most of the plastic types were optically identified by the former. Finally, the most important conclusion to be drawn is the influence of the shape of the MP particles on their sedimentation as a result of their complex light scattering. We clearly see from this set of samples that the time evolution of the speckles is a very good indicator of the plastic shape, which is convoluted with the density of the plastics, i.e., their types. These complementary observations justify the use of several optical methods.

## 4. Conclusions

Microplastics extracted from wastewater sludge and suspended in ethanol were investigated using Raman microscopy and transmission measurements, as well as novel laser-based techniques. It was demonstrated that combining Raman and transmission techniques is a promising tool in the quantitative identification and validation of common microplastics, such as polypropylene (PP), polyethylene terephthalate (PET), polystyrene (PS), and polyethylene (PE), that are present in processed wastewater sludge samples. Moreover, the results also show that basic laser-based measurement can be a useful technique for qualitatively studying the dynamic properties of microplastics based on their complex light-scattering behavior. Additionally, the results indicate that the density of MPs plays a vital role in the dynamic properties and can affect their detection in natural environments. This study represents a step towards the development of a multifunctional optical sensor for the detection of microplastics and their dynamic properties of light scattering in natural environments based on their spectral features and speckle metrology. Such a device could serve as a qualitative control in wastewater treatment processes by monitoring MPs after treatment. A lack of reliable methods for the detection of complex shapes, which are most common in wastewater, appears in current research on MPs. Most of the optical methods used so far are mainly based on Mie light scattering, which is valid only for spherical particles. Thus, the development of a multifunctional optical sensor that employs both spectroscopic and non-spectroscopic methods, as described in this work, could be useful in the monitoring of micro-or nanoplastics and their essential characteristics, such as complex shapes, sizes, and sedimentation in situ in real field conditions. Studies are ongoing in order to establish further correlations between the proposed techniques and conventional methods and to integrate different optical techniques for the development of a multifunctional optical sensor for the identification of MPs in aquatic environments.

## Figures and Tables

**Table 1:** Sequence of reagents used to purify the microplastic samples according to Löder et al. (2017) [17].

Step	Reagent	Incubation Temperature and Time
**1**	Hydrogen peroxide 30%	60 °C, 1 d
**2**	Protease in Tris-HCl buffer (1:5, pH = 9)	50 °C, 1 d
**3**	Lipase in Tris-HCl buffer (1:20, pH = 9)	40 °C, 1 d
**4**	Cellulase in NaOAc buffer (1:4, pH = 5)	40 C, 3 × 1 d
**5**	Hydrogen peroxide 30%	60 C, 1 d
**6**	Chitinase in NaOAc (1:20, pH = 5)	37 °C, 1 d
**7**	Hydrogen peroxide 30%	60 °C, 1 d
**8**	Density separation with ZnCl_2_ (ρ = 1.6–1.8 g.cm^−3^)	Room temperature, 1–3 d

**Table 2:** Identified MP types and quantities per dry weight (DW) from Raman microscopy and average densities in pristine form. MPs g^−1^ DW: Microplastics per gram of dry weight. MPs/mL: Microplastics per millilitre ethanol. Particle (P) or fiber (F).

	Sample A		Sample B		Sample C		
**Dry Weight [g]**	11.5		13.8		13.9		
**Proportion of DW analyzed with Raman [%]**	0.9		0.9		2		
**Fibers counted**	6		4		15		
**Particles counted**	8		7		6		
**MPs g^−1^ DW**	130		85		85		
**MPs/mL**	19		15		17		
**MP types**	**F**	**P**	**F**	**P**	**F**	**P**	**Density in pristine form [g cm^−3^]**
**PP**	0	3	0	1	0	3	0.85
**PE**	1	0	0	1	3	0	0.94
**PET**	5	1	4	2	12	1	1.38
**PS**	0	1	0	0	0	1	1.05
**POM**	0	3	0	3	0	1	1.42
